# The relationship between family function and behavioral decision-making in patients with gestational diabetes mellitus: a multiple mediation model and cross-lagged analysis of pregnancy-related anxiety and hope

**DOI:** 10.3389/fmed.2026.1838381

**Published:** 2026-06-17

**Authors:** Rui Li, Jing Peng, Yun Miao, Qinhui Yu, Hong Song

**Affiliations:** 1 Chronic Disease Management Center, The Second Affiliated Hospital of Xuzhou Medical University, Xuzhou, Jiangsu, China; 2 Department of Nursing, The Second Affiliated Hospital of Xuzhou Medical University, Xuzhou, Jiangsu, China

**Keywords:** blood glucose management behavioral decision-making, chain mediation, cross-lagged analysis, family function, gestational diabetes mellitus, hope, longitudinal study, pregnancy-related anxiety

## Abstract

**Aim:**

This study aimed to explore the chain mediating effect and cross-lagged associations between pregnancy-related anxiety and hope in the relationship between family function (FF) and blood glucose management behavioral decision-making (BDM) among patients with gestational diabetes mellitus (GDM). This study also aimed to provide insights into enhancing self-management efficacy and optimizing maternal and infant outcomes.

**Methods:**

A convenience sample of 245 GDM patients was selected. Surveys were conducted using the Pregnancy-related Anxiety Questionnaire (PAQ), the Herth Hope Index (HHI), the Family Adaptation, Partnership, Growth, Affection, and Resolve (APGAR) Index, and the Blood Glucose Management Decision-Making Behavior Questionnaire at three time points: 24–28 weeks of gestation (T1), 32 weeks of gestation (T2), and 36–42 weeks of gestation (T3). Statistical analyses included equivalence testing and cross-lagged modeling.

**Results:**

A total of 220 valid questionnaires were collected. Equivalence testing indicated that all variables met the requirements for measurement invariance, ensuring cross-temporal comparability of the tools. Cross-lagged path analysis revealed that T1 family function significantly predicted T2 pregnancy-related anxiety (*β* = −0.26, *p* < 0.01), T2 hope (*β* = 0.18, *p* < 0.01), and T2 behavioral decision-making (*β* = 0.24, *p* < 0.001). T1 pregnancy-related anxiety significantly predicted T2 hope (*β* = −0.11, *p* < 0.01) and T2 behavioral decision-making (*β* = −0.28, *p* < 0.001). T2 family function positively predicted T3 hope (*β* = 0.18, *p* < 0.001), while T2 pregnancy-related anxiety negatively predicted T3 behavioral decision-making (*β* = −0.25, *p* < 0.001). T2 hope positively predicted T3 behavioral decision-making (*β* = 0.18, *p* < 0.001), and T2 behavioral decision-making positively predicted T3 family function (*β* = 0.11, *p* < 0.01). Bootstrap analysis demonstrated significant indirect effects of T2 pregnancy-related anxiety (*β* = 0.065, *p* < 0.01) and T2 hope (*β* = 0.032, *p* < 0.01) in the prediction of T3 behavioral decision-making by T1 family function.

**Conclusion:**

Clinical practice should develop family-centered intervention models, assess family function, screen for anxiety, and enhance hope through psychological support and health education to promote positive behavioral decision-making and improve blood glucose control.

## Introduction

Gestational diabetes mellitus (GDM) is a growing public health concern worldwide. Its effective management relies not only on standardized clinical treatment but also on close association with patients’ self-management behaviors (1). Behavioral decision-making is the core process through which patients actively participate in blood glucose monitoring, dietary adjustments, and regular exercise, directly impacting disease control outcomes (2, 3). However, clinical observations indicate significant heterogeneity in the behavioral decision-making levels of GDM patients, with some struggling to maintain effective self-management strategies, leading to increased blood glucose fluctuations and a higher risk of adverse pregnancy outcomes (4).

Previous research has suggested that family function, as a key social support resource, can significantly influence the behavioral decision-making of GDM patients (5). Good family communication, emotional support, and collaborative problem-solving abilities help patients establish positive disease coping models (6). There is a complex bidirectional association between reproductive disorders and metabolic diseases. Existing evidence has indicated that reproductive endocrine diseases, including GDM, are often accompanied by hyperactivation of the hypothalamic–pituitary–adrenal (HPA) axis and a state of chronic low-grade inflammation. This not only exacerbates insulin resistance but also induces psychological stress responses, such as anxiety and depression (7). Pregnant women undergo significant physiological, psychological, and social role changes. A GDM diagnosis can further induce pregnancy-related anxiety. Such negative emotions may deplete cognitive resources, hindering the ability to make healthy decisions (8). Hope, a key concept in positive psychology, reflects a patient’s confidence in disease outcomes and motivation to achieve health goals. Patients with higher levels of hope typically exhibit greater initiative in self-management (9).

The majority of current studies on the relationships between family function, anxiety, hope, and behavioral decision-making in GDM patients are based on cross-sectional data, making it difficult to infer causal order and temporal evolution (10). Furthermore, it remains to be verified whether family function can indirectly promote behavioral decision-making by alleviating anxiety and enhancing hope. Therefore, this study uses a cross-lagged panel analysis to construct a chain mediation model, exploring the longitudinal mediating roles of pregnancy-related anxiety and hope between family function and behavioral decision-making in GDM patients. The goal is to clarify how family function promotes positive behavioral decision-making by alleviating anxiety and boosting hope, ultimately enhancing self-management efficacy and optimizing maternal and infant outcomes.

## Research objects and methods

### Research objects

From June 2024 to August 2025, convenience sampling was used to select 245 patients with gestational diabetes mellitus who attended regular prenatal examinations at the outpatient department of a Class III Grade A hospital in Xuzhou as participants. Ethical approval was obtained from the Office of Medical Ethics of the Second Hospital of Xuzhou Medical University before the study began (batch number: [2024] 050011). The inclusion criteria were as follows: (1) patients with gestational diabetes (11), (2) patients aged 18 years and above, and (3) patients with basic communication skills who all provided signed informed consent. The exclusion criteria were as follows: (1) patients with a previous history of gestational diabetes mellitus, (2) patients with severe heart failure and other organic diseases, and (3) patients with severe complications during pregnancy. The withdrawal criteria were as follows: (1) premature birth, stunting, or death of the fetus due to various reasons during pregnancy or (2) missing data from the questionnaire on more than one occasion. The cross-lagged model was used to test the chain mediating effect (12). The sample size met the minimum requirement for complex models, and the statistical power was verified via a Monte Carlo simulation. Considering the high attrition rate typical of longitudinal studies, a dropout rate of 15% was assumed based on previous research (13). Consequently, the initial sample size was calculated as n = 200/0.85 ≈ 235, leading to the enrollment of 245 patients in this study.

### Survey tools

#### Questionnaire for basic information

The questionnaire was self-compiled by the research team. It includes demographic data—age, occupation, residence, living style, education level, and family monthly income—and disease-related data (number of pregnancies and mode of conception) among GDM patients.

#### Pregnancy-related anxiety scale

The Pregnancy-related Anxiety Questionnaire (PAQ) was developed by Xiao et al. (14). Each item was scored using the Likert 4-point scoring method, ranging from 1 to 4 points, from “not worried” to “always worried.” The total score ranged from 13 to 52 points, and the higher the score, the higher the level of pregnancy-related anxiety. The Cronbach’s *α* coefficient of the scale was 0.81. The Cronbach’s α coefficients of the scale in this study were 0.800, 0.815, and 0.863.

#### Herth Hope scale

The Herth Hope Index (HHI) was developed by Herth (15) and translated into Chinese by Herth (15) and Zhao et al. (16). The HHI includes three dimensions: positive attitude toward reality and the future (4 items), taking positive action (4 items), and maintaining close relationships with others (4 items), with a total of 12 items. Each item was scored using a Likert 4-point scale, ranging from “completely disagree” to “completely agree,” scored from 1 to 4 points; items 3 and 6 were reverse-scored. The total score ranged from 12 to 48 points, and the higher the score, the higher the individual’s hope. The Cronbach’s *α* coefficient was 0.88. The Cronbach’s α coefficients of the scale in this study were 0.890, 0.825, and 0.848.

#### APGAR index

The Family Adaptation, Partnership, Growth, Affection, and Resolve (APGAR) Index was developed by Smilkstein et al. (17) and translated into Chinese by the Chinese scholar Lu and Liu et al. (18). The scale contains five items, namely adaptability, growth, cooperation, affection, and affinity. The Likert 3-point scoring method was used, ranging from “almost rarely” to “often.” The total score ranged from 0 to 10, and the higher the score, the higher the level of family function. The Cronbach’s *α* coefficients of the scale in this study were 0.833, 0.905, and 0.830.

#### GDM blood glucose management decision-making behavior questionnaire

The Gestational Diabetes Mellitus Blood Glucose Management Decision-Making Behavior Questionnaire (GBM-MDMBQ) was compiled by Huang et al. (19). The questionnaire contains 15 items, including outpatient follow-up, diet management, exercise management, blood glucose self-monitoring, drug therapy, and other items. The items were scored using the Likert 5-point scale, ranging from 1 to 5 points from “completely inconsistent” to “completely consistent,” and the total score ranges from 15 to 75 points. The higher the score, the better the patient’s blood glucose management decision-making behavior. The content validity index of the questionnaire was 1.000, and the Cronbach’s *α* coefficient was 0.905. The Cronbach’s α coefficients of the scale in this study were 0.855, 0.900, and 0.848.

### Questionnaire recovery and quality control

The questionnaire survey was conducted after obtaining informed consent from the patients and approval from the hospital ethics committee. The follow-up time was determined through a literature review (20). The follow-up time points were 24–28 weeks of gestation (T1), 32 weeks of gestation (T2), and 36–42 weeks of gestation (T3). The present study selected gestational weeks 24–28 (T1), 32 (T2), and 36–42 (T3) as longitudinal follow-up time points, primarily based on physiological metabolic changes during pregnancy, patterns of psychological fluctuation, and standard clinical practice.

First, 24–28 weeks of gestation represent the conventional window recommended by the International Association of Diabetes in Pregnancy Study Groups (IADPSG) and national guidelines for the screening and diagnosis of GDM (21). At this stage, the secretion of placental insulin-antagonistic hormones increases significantly, making glucose metabolism abnormalities most detectable. Therefore, it serves as the most appropriate starting point for assessing baseline family functioning, psychological status, and behavioral decision-making post-diagnosis.

Second, 32 weeks of gestation falls within the transition phase of the third trimester. As pregnancy progresses, physical burdens intensify, and concerns regarding delivery often peak, leading to marked fluctuations in pregnancy-related anxiety. This time point aligns with routine prenatal visit schedules, making it suitable for capturing the mediating psychological states and behavioral patterns during this “high-pressure accumulation phase.”

Finally, weeks 36–42 represent the pre-delivery/full-term stage. As parturition approaches, psychological stress, including anxiety and fluctuations in hope, and self-management behavioral decision-making undergo final adjustments. This period is critical for observing the dynamic evolution and outcomes of these variables during pregnancy and facilitates a seamless connection with postpartum follow-up.

Patients with gestational diabetes mellitus were investigated by a specialist in chronic diabetes disease management via the follow-up system using the PAQ, HHI, APGAR index, and GDM Blood Glucose Management Decision-Making Behavior Questionnaire. The survey methods included online questionnaires distributed through the follow-up system, telephone interviews, and face-to-face surveys.

Data collection and entry were performed by two researchers. One was responsible for data collection, and the other independently checked the data to ensure the accuracy of data entry. To improve patient participation, patients who completed the surveys from T1 to T2 were given a birth gift package.

### Statistical methods

Enumeration data were expressed as counts and percentages (%), and measurement data were expressed as means ± standard deviations. Pearson’s correlation analysis was used to examine the correlations among the variables, and the Harman single-factor method was used to test for common method variance. Confirmatory factor analysis (CFA) was used to assess the reliability and validity of the scale, as well as the goodness-of-fit of the measurement model.

Mplus 8.3 software was used to construct the chain mediation model via Hayes’ PROCESS macro program. The non-parametric percentile bootstrap method with bias correction was applied to conduct 5,000 repeated samplings to test the multiple mediating effects of pregnancy-related anxiety and hope between family function and behavioral decision-making.

Mplus 8.3 software was also used for the measurement equivalence test and cross-lagged model analysis. The cross-lagged model was used to analyze the temporal causality between variables. By comparing the standardized coefficients of different lagged paths, the cross-time predictive effect of pregnancy-related anxiety on hope was clarified while controlling for baseline effects.

To rule out potential confounding effects from demographic and clinical characteristics, this study incorporated maternal age, educational level, monthly household income, parity, conception method, and fasting blood glucose levels at GDM diagnosis as covariates when constructing the cross-lagged models and performing bootstrap mediation analyses. By controlling for the effects of these variables at baseline (T1), we aimed to ensure that the observed longitudinal associations among family function, psychological variables, and behavioral decision-making were not driven by these stable individual characteristics.

A fitting index with a comparative fit index (CFI) of > 0.900, a Tucker–Lewis Index (TLI) > of 0.900, and a root mean square error of approximation (RMSEA) of <0.08 indicated that the cross-lagged model was well-fitted, and the test level was set at *α* = 0.05.

## Results

### General demographic data

A total of 25 participants were lost to follow-up at T2–T3, and 220 valid questionnaires were obtained (effective rate: 89.80%). Comparisons of general demographic and clinical characteristics between the attrited participants (*n* = 25) and the final sample (*n* = 220) were conducted, and no statistically significant differences were observed (*p* > 0.05). The participants were aged 22–46 years, with an average age of (30.42 ± 2.23) years. The specific information is shown in Table 1.

**Table 1:** General information of the participants (*n* = 220).

Items	*n*	Percentage (%)
Age (years)		
<30	128	58.18
30 ~ 40	72	32.73
>40	20	9.09
Career		
Public institution	31	14.09
Professional technicians	38	17.27
Staff/staff	121	55.00
No regular occupation	30	13.64
Place of residence		
Rural	104	47.27
Towns	116	52.73
Style of residence		
Couple residence	152	69.09
Live with your parents/in-laws	68	30.91
Monthly household income (yuan)		
<5,000	61	27.73
5,000 ~ 9,999	95	43.18
>10,000	64	29.09
Education level		
Junior high school and below	47	21.36
High school	82	37.27
College and above	91	41.37
Number of pregnancies		
1	144	65.45
≥2	76	34.55
Method of conception		
Conceived naturally	179	81.36
Artificial insemination	41	18.64

### Common method bias test

The common method deviation test was conducted on the three sets of data using the Harman single-factor test. The variances of the three measurements were 15.34, 21.67, and 28.05%, respectively, which were all below the 40% critical value (22). Therefore, there was no evidence of serious common method bias in this study.

### Scores and correlation analysis of pregnancy-related anxiety, hope, family function, and behavioral decision-making in patients with gestational diabetes mellitus

The scores of pregnancy-related anxiety, family function, and behavioral decision-making of patients with gestational diabetes mellitus showed significant dynamic changes over time (*p* < 0.001). In contrast, the hope score showed no significant change over time (*p* = 0.051), as shown in Table 2.

**Table 2:** Scores of pregnancy-related anxiety, hope, family function, and behavioral decision-making in patients with gestational diabetes mellitus (*n* = 220, score, *х* ± *ѕ*).

Items	PAQ	HHI	APGAR	GBM-MDMBQ
T1	30.55 ± 4.17	37.65 ± 2.46	6.33 ± 0.96	42.98 ± 5.71
T2	29.21 ± 3.44	38.91 ± 2.45	6.95 ± 0.94	46.47 ± 6.13
T3	28.16 ± 3.74	38.52 ± 2.47	6.10 ± 0.92	43.88 ± 4.66
*F*-score	18.423	3.007	16.030	14.074
*p*-value	<0.001	0.051	<0.001	<0.001

The scores of hope, family function, and behavioral decision-making were positively correlated during the T1–T3 stage. Moreover, they were all negatively correlated with the score of pregnancy-related anxiety, and the correlations were significant (*p* < 0.05), as shown in Table 3.

**Table 3:** Correlation coefficient matrix of variables at three time points (*r* value, *n* = 220).

Items	①	②	③	④	⑤	⑥	⑦	⑧	⑨	⑩	⑪	⑫
① PAQ T1	1											
② HHI T1	−0.413^**^	1										
③ APGAR T1	−0.390^**^	0.558^**^	1									
④ GBM-MDMBQ T1	−0.453^**^	0.489^**^	0.480^**^	1								
⑤ PAQ T2	0.560^**^	−0.300^*^	−0.377^**^	−0.460^**^	1							
⑥ HHI T2	−0.393^**^	0.486^**^	0.459^**^	0.354^*^	−0.393^**^	1						
⑦ APGAR T2	−0.301^*^	0.444^**^	0.468^**^	0.475^**^	−0.372^**^	0.449^**^	1					
⑧ GBM-MDMBQ T2	−0.354^**^	0.407^**^	0.437^**^	0.457^**^	−0.409^**^	0.465^**^	0.491^**^	1				
⑨ PAQ T3	0.453^**^	−0.277^*^	−0.502^**^	−0.304^*^	0.447^**^	−0.453^**^	−0.390^**^	−0.453^**^	1			
⑩ HHI T3	−0.284^*^	0.359^**^	0.307^**^	0.379^**^	−0.372^**^	0.414^**^	0.406^**^	0.474^**^	−0.370^**^	1		
⑪ APGAR T3	−0.265^*^	0.320^**^	0.403^**^	0.388^**^	−0.355^**^	0.421^**^	0.446^**^	0.446^**^	−0.389^**^	0.407^**^	1	
⑫ GBM-MDMBQ T3	−0.308^*^	0.355^**^	0.397^**^	0.401^**^	−0.397^**^	0.435^**^	0.400^**^	0.412^**^	−0.376^**^	0.441^**^	0.423^**^	1

* denotes *P* < 0.05, ** denotes *P* < 0.01.

### Equivalence model of three measurements of pregnancy-related anxiety, hope, family function, and behavioral decision-making in patients with gestational diabetes mellitus

This study used Mplus 8.3 to construct configural, metric, and scalar invariance models. First, a baseline configural invariance model (Model 1) was established, specifying identical configurations for observed indicators of the same latent variable across the three time points while allowing residual errors of identical items to correlate over time; all other parameters were freely estimated. Building on the configural model, a metric invariance model (Model 2) was constructed by constraining factor loadings to be equal across time. Finally, a scalar invariance model (Model 3) was established by adding equality constraints on item intercepts across waves. Measurement invariance was evaluated using the criteria of ΔCFI ≤0.01, ΔTLI ≤0.01, and ΔRMSEA ≤0.015 (23). By comparing changes in model fit indices under different constraint conditions, we verified the longitudinal measurement invariance of the instruments across the three assessments. As shown in Table 4, all variables satisfied the requirements for measurement invariance, indicating that the measurement tools were cross-temporally comparable, subsequent justifying cross-lagged path analyses.

**Table 4:** Equivalence model of three measurements of pregnancy-related anxiety, hope, family function, and behavioral decision-making in patients with gestational diabetes mellitus.

Variables	Model	*χ^2^*	*df*	CFI	TLI	RMSEA (95%CI)	SRMR	∆CFI	∆TLI	∆RMSEA
Pregnancy-related anxiety	Model 1	790.536	375	0.944	0.931	0.047 ([0.043, 0.052])	0.065	–	–	–
	Model 2	806.691	383	0.945	0.929	0.048 ([0.043, 0.052])	0.066	0.003	0.005	0.002
	Model 3	821.543	394	0.942	0.934	0.047 ([0.042, 0.053])	0.066	0.003	0.004	0.002
Hope	Model 1	1013.814	482	0.964	0.949	0.048 ([0.044, 0.053])	0.077	–	–	–
	Model 2	1041.835	494	0.962	0.968	0.048 ([0.044, 0.053])	0.078	0.002	0.004	0.001
	Model 3	1058.306	508	0.960	0.950	0.047 ([0.043, 0.052])	0.079	0.002	0.004	0.001
Family function	Model 1	147.753	68	0.936	0.926	0.051 ([0.040, 0.061])	0.036	–	–	–
	Model 2	159.103	75	0.931	0.935	0.050 ([0.040, 0.061])	0.039	0.004	0.005	0.002
	Model 3	170.125	81	0.930	0.928	[0.040, 0.060]	0.040	0.003	0.005	0.002
Behavioral decision-making	Model 1	397.072	158	0.951	0.937	0.057 ([0.050, 0.064])	0.046	–	–	–
	Model 2	410.020	167	0.950	0.940	0.055 ([0.050, 0.061])	0.047	0.002	0.003	0.001
	Model 3	434.574	175	0.948	0.939	0.056 ([0.049, 0.063])	0.049	0.002	0.003	0.001

*χ*^2^ is chi-square, df is degrees of freedom, CFI is comparative fit index, TLI is Tucker–Lewis index, RMSEA is root mean square of approximation, and SRMR is standardized root mean square residual.

### Cross-lagged analysis of the chain mediating effect of pregnancy-related anxiety and hope between family function and behavioral decision-making in patients with gestational diabetes mellitus

#### Establishment and fitting of the model

Four cross-lagged models were established and tested to determine the best model for the longitudinal relationship of each variable involved. These included the baseline model (M1), which was the autoregressive path from T1 to T3. The one-way prediction model (M2) added, based on the baseline model, the path of family function to hope, pregnancy-related anxiety, and behavioral decision-making; the path of hope to pregnancy-related anxiety and behavioral decision-making; and the path of hope to behavioral decision-making. The one-way prediction model (M3) added, based on the baseline model, the path of pregnancy-related anxiety to family function; the path of hope to family function and pregnancy-related anxiety; and the path of behavioral decision-making to family function, pregnancy-related anxiety, and hope. The integrated model (M4) included all pathways from M1 to M3. Table 5 shows the fitting indices of the four models and the results of the model comparison.

**Table 5:** Model fitting and comparison results.

Models	*χ^2^*	df	CFI	TLI	RMSEA	∆*χ^2^* (df) vs. M1	∆*χ^2^* (df) vs. M4
M1	2255.213	520	0.935	0.922	0.093	–	–
M2	1246.137	386	0.972	0.968	0.041	1009.076 (184)^*^	523.758 (184)^*^
M3	1295.262	380	0.966	0.950	0.042	959.951 (182)^*^	572.883 (182)^*^
M4	722.379	204	0.987	0.958	0.022	–	–

M1 is the baseline model, M2 and M3 are one-way prediction models, and M4 is the integrated model; * denotes *P* < 0.01.

According to the model comparison results in Table 4, the baseline model (M1) was excluded because of insufficient fit. The comparative analysis indicated that the one-way prediction models M2 and M3 demonstrated significant improvement compared with M1 (∆CFI > 0.01); however, there was no evidence of equivalence between them. Compared with M2 and M3, the integrated model M4 further optimized the model fit index (∆CFI > 0.01). The fit indices for Model 4 indicated a good fit to the data: *χ*^2^/df = 3.541, CFI = 0.987, TLI = 0.958, and RMSEA = 0.022. Based on the comprehensive comparison of model fit indices, Model 4 was selected as the final analytical model.

#### Path analysis of the cross-lagged model

**Figure 1:**
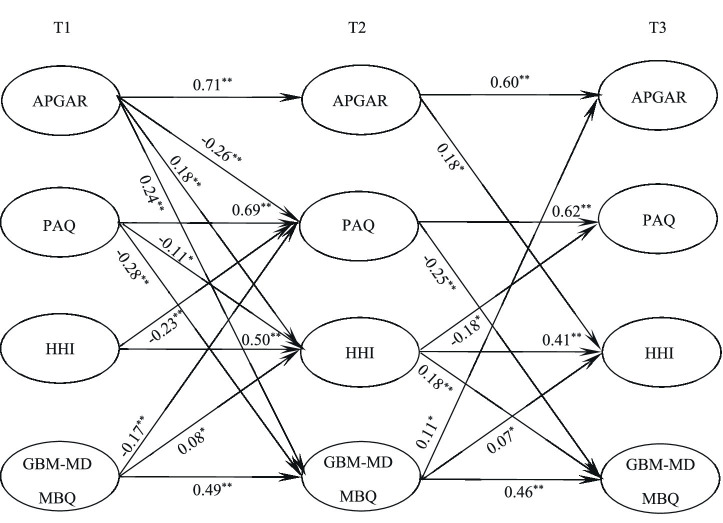
Cross-lagged path diagram of the chain mediation of pregnancy-related anxiety and hope between family functioning and behavioral decision-making in patients with gestational diabetes mellitus. Only significant standardized paths are presented. *p < 0.01, **p < 0.001.

The final integrated model (M4) was fitted with maternal age, educational level, monthly household income, parity, and baseline fasting blood glucose included as control variables. The results demonstrated that the path coefficients among the variables remained statistically significant even after accounting for the potential confounding effects of these covariates. Figure 1 shows the cross-lagged model of family function, pregnancy-related anxiety, hope, and behavioral decision-making. In this model, the autoregressive paths of each variable across T1–T3 were significant, and the variables at T1 and T2 could positively predict the variables at T2 and T3 (*p* < 0.01). Specific paths are presented in Table 6.

**Table 6:** Cross-lagged analysis of the chain mediating roles of pregnancy-related anxiety and hope between family function and behavioral decision-making in patients with gestational diabetes mellitus.

Path	*β*	95%CI
T1 family function → T2 pregnancy-related anxiety	−0.26**	−0.35 ~ −0.15
T1 family function → T2 hope	0.18**	0.10 ~ 0.27
T1 family function → T2 behavioral decision-making	0.24**	0.12 ~ 0.35
T1 pregnancy-related anxiety → T2 hope	−0.11*	−0.20 ~ −0.04
T1 pregnancy-related anxiety → T2 behavioral decision-making	−0.28**	−0.37 ~ −0.17
T2 family function → T3 Hope	0.18**	0.10 ~ 0.25
T2 pregnancy-related anxiety → T3 behavioral decision-making	−0.25**	−0.35 ~ −0.16
T2 hope → T3 behavioral decision-making	0.18**	0.11 ~ 0.25
T2 behavioral decision-making → T3 family function	0.11*	0.05 ~ 0.16
T1 family function → T2 pregnancy-related anxiety → T3 behavioral decision-making	0.065*	0.02 ~ 0.11
T1 family function → T2 Hope → T3 behavioral decision-making	0.032*	0.01 ~ 0.05

* indicates *P* < 0.01, **P < 0.001.

Family function at T1 significantly predicted pregnancy-related anxiety (*β* = −0.26, *p* < 0.001), hope at T2 (*β* = 0.18, *p* < 0.01), and behavioral decision-making at T2 (*β* = 0.24, *p* < 0.001). Pregnancy-related anxiety at T1 significantly predicted hope at T2 (*β* = −0.11, *p* < 0.01) and behavioral decision-making at T2 (*β* = − 0.28, *p* < 0.001).

Family function at T2 significantly positively predicted hope at T3 (*β* = 0.18, *p* < 0.001), pregnancy-related anxiety at T2 significantly negatively predicted behavioral decision-making at T3 (*β* = −0.25, *p* < 0.001), and hope at T2 significantly positively predicted behavioral decision-making at T3 (*β* = 0.18, *p* < 0.001). T2 behavioral decision-making significantly positively predicted T3 family function (*β* = 0.11, *p* < 0.01).

Bootstrap analysis showed that T2 pregnancy-related anxiety (*β* = 0.065, *p* < 0.01) and T2 hope (*β* = 0.032, *p* < 0.01) had significant indirect effects on T1 family function in the prediction of T3 behavioral decision-making. These results indicated that pregnancy-related anxiety and hope had a longitudinal mediating effect on the mechanism of family function on behavioral decision-making.

### Sensitivity analysis

To address the limitation that path coefficients in the traditional Cross-Lagged Panel Model (CLPM) may confound stable between-person trait differences with dynamic within-person fluctuations (24) and to verify the robustness of the core hypotheses, we further constructed a Random Intercept Cross-Lagged Panel Model (RI-CLPM). By incorporating random intercepts for family function, pregnancy-related anxiety, hope, and behavioral decision-making, the RI-CLPM isolates stable individual differences from the cross-lagged paths. Crucially, in addition to controlling for these random intercepts representing stable between-person traits, time-invariant covariates, including maternal age, educational level, household income, parity, conception method, and baseline fasting blood glucose, were also included in the model. The results revealed that the direction and significance levels of the core paths (i.e., T1 family function → T2 pregnancy-related anxiety, T2 pregnancy-related anxiety → T3 behavioral decision-making) remained largely unchanged after accounting for both stable traits and demographic controls (Table 7). This indicates that the predictive effects observed are not merely attributable to inherent, stable personal characteristics but rather reflect the genuine dynamic evolution of these variables over time.

**Table 7:** Comparison of path coefficients between the CLPM and RI-CLPM (standardized β).

Path	*β*	RI-CLPM (β)	Change in significance
Within-person effects			
T1 FF → T2 PRAS	−0.26**	−0.22**	No
T1 FF → T2 hope	0.18**	0.15*	No
T1 PRAS → T2 hope	−0.11*	−0.09*	No
T2 PRAS → T3 BDM	−0.25**	−0.21**	No
T2 Hope → T3 BDM	0.18**	0.16**	No
Between-person effects			
RI_FF ↔ RI_PRAS	–	−0.48*	–
RI_FF ↔ RI_Hope	–	0.52*	–
RI_PRAS ↔ RI_BDM	–	−0.39*	–
Model fit			
CFI	0.987	0.981	Acceptable
RMSEA	0.022	0.028	Acceptable

FF, family function; PRAS, pregnancy-related anxiety; BDM, behavioral decision-making. * indicates *P* < 0.01, **P < 0.001.

## Discussion

The results of this study revealed that there were significant dynamic changes in the psychological state and the behavior pattern of patients with gestational diabetes mellitus during the second and third trimesters of pregnancy and postpartum. The scores of pregnancy-related anxiety, family function, and blood glucose management behavioral decision-making showed significant dynamic changes over time. The hope level fluctuated but did not reach statistical significance, which may be because the patients experienced a period of adaptation in the early stage after diagnosis. As the gestational age increased and their understanding of the disease deepened, their psychological stress response gradually stabilized (25).

According to Family Systems Theory, family function does not exist in isolation. Instead, it constitutes an entire system that interacts with individual mental states and health behaviors, and changes in any link affect other parts through a feedback mechanism (26). This study is the first to longitudinally substantiate the temporal dynamics of this interaction: family functioning not only predicted subsequent behavioral decision-making, but prior behavioral decision-making also positively predicted later family functioning (*β* = 0.11, *p* < 0.01). These findings challenge the traditional conceptualization of the family as a static backdrop and instead reveal a reciprocal gain cycle between the family system and the individual. Specifically, when patients adhere to effective glycemic management decisions, they often yield positive health outcomes (e.g., stable blood glucose levels). This success not only alleviates disease-related conflicts and stress within the family but also conveys positive coping signals to other members, thereby enhancing family cohesion and adaptability. This bidirectional facilitative relationship suggests that interventions should extend beyond merely providing family support to improve patient behavior; rather, equal attention should be paid to how enhancing patient self-management can, in turn, foster greater family harmony. Further correlation analysis showed that from T1 to T3, the scores of hope, family function, and behavioral decision-making were significantly positively correlated, and all of them were significantly negatively correlated with pregnancy-related anxiety scores. This indicates that there was a synergistic effect among the patients’ psychological resilience, family support network, and self-management ability during the course of gestational diabetes mellitus, thereby forming a benign interaction mechanism.

Pregnancy-related anxiety may interfere with and weaken the formation of this mechanism, which was also confirmed in the study of Xing et al. (27). Xu et al. (32) showed that high anxiety levels undermine patients’ sense of hope, thereby reducing their ability to use family support and ultimately preventing them from engaging in active glycemic management behaviors (28). GDM represents a prototypical disorder of metabolic adaptation during pregnancy, characterized not only by pathophysiological alterations in insulin secretion and action but also by pronounced systemic inflammation and activation of the hypothalamic–pituitary–adrenal (HPA) axis. A bidirectional amplification effect exists between such metabolic dysregulation and psychological stress. Recent reviews have explicitly highlighted that hyperglycemia during pregnancy exacerbates the psychosocial burden on pregnant individuals via pathways involving chronic inflammation and oxidative stress, whereas persistent psychological stress further deteriorates metabolic control, thereby establishing a vicious cycle (29). In the present study, family functioning not only improved psychological adaptation by alleviating pregnancy-related anxiety and enhancing hope but also potentially optimized the glycemic metabolic milieu by attenuating excessive HPA axis activation. This finding provides dual physiological and psychological rationales for understanding the multidimensional benefits of family support in GDM management.

These findings suggest that, while conducting routine blood glucose monitoring and diet guidance for patients with gestational diabetes mellitus, clinical medical staff must place great emphasis on psychosocial interventions. They should incorporate pregnancy-related anxiety screening into the routine nursing process, relieve patients’ anxiety through cognitive behavioral therapy or mindfulness training, and actively mobilize family members to participate. Strengthening the support role of family function can improve the hope level of patients, stimulate their internal motivation, encourage them to adopt scientific blood glucose management behaviors, and ultimately ensure the safety of mother and child and optimize pregnancy outcomes.

The results of this study indicate that family function in the second trimester of pregnancy has a significant negative association with pregnancy-related anxiety during childbirth and a positive association with hope levels and behavioral decision-making. This suggests that, in the process of gestational diabetes management, a well-established family support system can help reduce the psychological burden of patients and enhance their confidence in coping with the disease through emotional care and substantial assistance. It also promotes positive self-management behavior, highlighting the crucial role of family function in the early intervention of the disease, which was also confirmed in the study by Zhang et al. (5).

According to the Conservation of Resources Theory, a high level of anxiety significantly consumes patients’ psychological resources, and family function, as an external resource, can timely supplement the depletion (30). The high level of pregnancy-related anxiety in the second trimester of pregnancy can significantly and negatively predict hope and behavioral decision-making during the waiting period, reflecting that anxiety not only impairs the patient’s current psychological state but may also continuously weaken their psychological resilience and initiative to participate in disease management. Therefore, in clinical study, we should pay attention to the long-term effects of this phenomenon. From the perspective of metabolic adaptation, pregnancy constitutes an endocrinologically intricate state requiring precise regulatory control; the onset of GDM signifies a decompensation in the maternal response to escalating energy demands. Such metabolic strain may further amplify psychological stress responses. Anxiety and hope, representing opposing poles of psychological adaptation, likely play pivotal roles in modulating maternal responses to metabolic challenges (29). The chain mediation findings of this study suggest that family functioning not only improves behavioral decision-making through the direct provision of material and emotional support but may also attenuate inflammatory responses and hormonal dysregulation by modulating psychological stress pathways. Consequently, this fosters a virtuous cycle linking metabolic and psychological adaptation.

Further analysis showed that family function during the awaiting-childbirth period could positively predict postpartum hope, while anxiety during the awaiting-childbirth period negatively predicted postpartum behavioral decision-making. At the same time, hope and behavioral decision-making during the awaiting-childbirth period had positive predictive effects on postpartum behavioral decision-making and family function, respectively, indicating that the psychological and behavioral characteristics in the third trimester of pregnancy have continuous effects on postpartum outcomes (31). There may be a mutually reinforcing relationship between behavioral decision-making and family function.

The bootstrap mediating effect test confirmed that anxiety and hope play a longitudinal mediating role between family function and behavioral decision-making, indicating that family function can indirectly affect patients’ self-management behavior by regulating their psychological state. This mediating pathway likely extends beyond a simple linear cascade. During the second trimester, robust family functioning fostered positive behavioral decision-making in the third trimester by alleviating anxiety and enhancing hope. Notably, this improved behavioral decision-making (T2) reciprocally reinforced family functioning in the postpartum period (T3). This suggests that anxiety and hope act as the “ignition switch” in the causal chain linking family functioning to behavior, whereas behavioral decision-making itself serves as the “stabilizer” that sustains long-term family resilience. In clinical practice, a family-centered comprehensive management model should be established, and family function assessment and targeted support should be initiated in the second trimester of pregnancy. By alleviating anxiety and cultivating hope, patients’ self-management ability can be improved, and maternal and infant outcomes can be enhanced.

### Limitations

This study has several limitations. First, the sample source was concentrated in a single region, which may lead to bias in the assessment results of family function due to regional cultural differences. In the future, multi-center and large-sample studies are needed to further verify the generalizability of the conclusions.

Second, the evaluation of behavioral decision-making on blood glucose management in the studies primarily relies on patient-reported scales, which have a certain risk of recall bias. In the future, objective indicators such as continuous glucose monitoring can be combined to more accurately investigate the actual effect of behavioral decision-making.

In addition, although this study reveals the chain mediating effect of pregnancy-related anxiety and hope between family function and behavioral decision-making, it cannot completely exclude the influence of potential confounding factors such as external social support and disease cognition. The robustness of this mediating effect still needs to be verified in follow-up studies.

## Conclusion

There is a mutual predictive relationship between family function and blood glucose management behavioral decision-making in patients with gestational diabetes mellitus. Pregnancy-related anxiety and hope play a longitudinal mediating role in the mechanism through which family function influences blood glucose management behavioral decision-making.

It is recommended to construct a family-centered intervention model initiated in the second trimester, focusing on identifying family support deficits and delivering tailored strategies (e.g., family education and involving family members in dietary planning). Integrating anxiety screening into routine prenatal visits and offering prompt psychological support (including CBT) are also advised. Additionally, fostering peer support groups can enhance patients’ hope, ultimately promoting proactive behavioral decision-making for glycemic control and establishing a beneficial feedback loop.

## Data Availability

The original contributions presented in the study are included in the article/supplementary material, further inquiries can be directed to the corresponding authors.
